# Review on Bortezomib Resistance in Multiple Myeloma and Potential Role of Emerging Technologies

**DOI:** 10.3390/ph16010111

**Published:** 2023-01-12

**Authors:** Gül Kozalak, İsmail Bütün, Erçil Toyran, Ali Koşar

**Affiliations:** 1 Faculty of Engineering and Natural Science, Sabancı University, Istanbul 34956, Turkey; 2 Center of Excellence for Functional Surfaces and Interfaces for Nano Diagnostics (EFSUN), Sabancı University, Istanbul 34956, Turkey; 3 Turkish Academy of Sciences (TÜBA), Çankaya, Ankara 06700, Turkey

**Keywords:** multiple myeloma, Bortezomib, multidrug resistance, nanoparticle, 3D cell culture, microfluidic, organ-on-chip

## Abstract

Multiple myeloma is a hematological cancer type. For its treatment, Bortezomib has been widely used. However, drug resistance to this effective chemotherapeutic has been developed for various reasons. 2D cell cultures and animal models have failed to understand the MM disease and Bortezomib resistance. It is therefore essential to utilize new technologies to reveal a complete molecular profile of the disease. In this review, we in-depth examined the possible molecular mechanisms that cause Bortezomib resistance and specifically addressed MM and Bortezomib resistance. Moreover, we also included the use of nanoparticles, 3D culture methods, microfluidics, and organ-on-chip devices in multiple myeloma. We also discussed whether the emerging technology offers the necessary tools to understand and prevent Bortezomib resistance in multiple myeloma. Despite the ongoing research activities on MM, the related studies cannot provide a complete summary of MM. Nanoparticle and 3D culturing have been frequently used to understand MM disease and Bortezomib resistance. However, the number of microfluidic devices for this application is insufficient. By combining siRNA/miRNA technologies with microfluidic devices, a complete molecular genetic profile of MM disease could be revealed. Microfluidic chips should be used clinically in personal therapy and point-of-care applications. At least with Bortezomib microneedles, it could be ensured that MM patients can go through the treatment process more painlessly. This way, MM can be switched to the curable cancer type list, and Bortezomib can be targeted for its treatment with fewer side effects.

## 1. Introduction

Multiple myeloma (MM) is a type of hematologic cancer characterized by the proliferation and propagation of a single variant of plasma B-cells [1]. Accumulating myeloma cells lead to bone destruction, anaemia, hypercalcaemia, and renal insufficiency [2]. MM represents 18% of hematologic malignancies, and the median age responding to diagnosis is 69 in the United States of America [3]. In its treatment, it is common to use combined therapy, which usually includes cytotoxic drugs. During recent years, proteasome inhibitors have provided the most important advantage in drug therapies [4]. Proteasome inhibitors interrupt the ubiquitin proteasome system. Thus, they inhibit the degradation of ubiquitinated proteins such as cyclin and cyclin-dependent kinase inhibitors that regulate the cell cycle, leading to an accumulation of proapoptotic factors, which results in triggering apoptosis [5]. Therefore, the discovery of these inhibitors provides a viable approach in selectively inducing apoptosis, reducing cell proliferation, and sensitizing tumour cells to conventional therapeutics [6]. The epoxy-ketone-based proteasome inhibitor Carfilzomib was approved by the United States Food and Drug Administration (FDA) in July 2012 for the treatment of patients with difficult-to-treat and relapsed MM. Carfilzomib has been proven to be effective for patients with MM who relapsed after Bortezomib therapy [7]. Ixazomib, the first oral proteasome inhibitor, has been approved by the United States and the European Union for the use with Lenalidomide and Dexamethasone in the treatment of MM patients who received prior chemotherapy [8].

Bortezomib is the first-class proteasome inhibitor approved for MM by the FDA and European Agency for Evaluation of Medicinal Products (EMEA). The advantage of Bortezomib is that it provides selective and reversible inhibition. Bortezomib’s inhibition was demonstrated to be effective in 60 human tumour cell lines, which were included in the NCI Therapy Development Program, and it was reported that bortozomib has different effects in different types of cancer (Table 1). Reversible inhibition of proteosome functions reached optimum anticancer activity (approximately 70%) in MM cell lines [9]. Bortezomib also reduced tumour growth in xenograft mouse models for multiple myeloma, adult T-cell leukaemia, lung, breast, prostate, pancreatic, head and neck, melanoma cancers [10].

The majority of chemotherapeutics cannot successfully pass through Phase 3 studies [23], which is due to the traditional cell culture and animal models used in preclinical studies [24]. As a result of advances in fabrication technology, cell culture systems have been moved from two-dimensional to three-dimensional platforms. On one hand, there are 2D cell cultures and high-cost mouse models that are insufficient to represent the tumour micro environment, while on the other hand there are 3D models, new generation microfluidic, and organ-on-chip devices that have evolved to investigate the angiogenesis, metastasis, and relationships with the tumour on a small chip. Thanks to this emerging technology, the preclinical trials of the prodrugs can be done, and distribution of the existing drugs can be made. The appropriate treatment method can be maintained by determining the resistance profile in the cells taken from the patients beforehand. In this review, we will first discuss the bortezomib-induced drug resistance mechanisms in multiple myeloma and then examine the role of emerging technologies such as 3D models, microfluidics, organ-on-chip devices, and nanoparticles in elucidating bortezomib resistance in multiple myeloma.

## 2. Mechanisms of Bortezomib Resistance in MM Cell

Bortezomib is the most effective chemotherapeutic drug used in the treatment of MM. This inhibitor is a dipeptide boronic acid analogue discovered in 1995 and is the premier in the class of chymotrypsin-like (CP) inhibitors. Bortezomib is a C-terminal boronic acid, and the boron atom is essential for inhibiting the proteasome activity because of its ability to specifically and tightly bind the β5 catalytic subunit. Boronates form tetrahedral adducts, which are further stabilized by a hydrogen bond between the N-terminal amino group of threonines and the hydroxyl groups of boronic acid. These bonds provide a higher influence for Bortezomib than other drugs developed for inhibition. It binds the proteasome with a high resolution, slowly dissociates, and provides a stable but reversible proteasome inhibition. However, after a while, Bortezomib resistance develops in patients. Multidrug resistance frequently occurs in multiple myeloma and is strongly associated with relapse of the disease. Bortezomib resistance was observed even in newly diagnosed MM patients who received treatment for the first time [25]. The survival of cancer cells as a result of removal of chemotherapeutics such as Bortezomib due to various reasons is defined as multi-drug resistance (MDR). MDR is the most prominent cause of the loss in effects of chemotherapeutics and constitutes an important issue in the treatment of cancer [26]. Various mechanisms have been suggested to explain the multidrug resistance in cancer cells. Increased drug excretion, decreased drug uptake, activation of detoxification systems, inhibition of apoptosis, alterations in cell cycle regulation factors, and changes in drug targets are among the causes [27]. Although almost all of these mechanisms are related to MM, many of them trigger each other. Therefore, it is impossible to consider resistance mechanisms in a completely independent fashion. The mechanisms of resistance by selecting those related to Bortezomib resistance in MM are summarized in Table 2.

### 2.1. Abnormal Drug Transport

**Figure 1:**
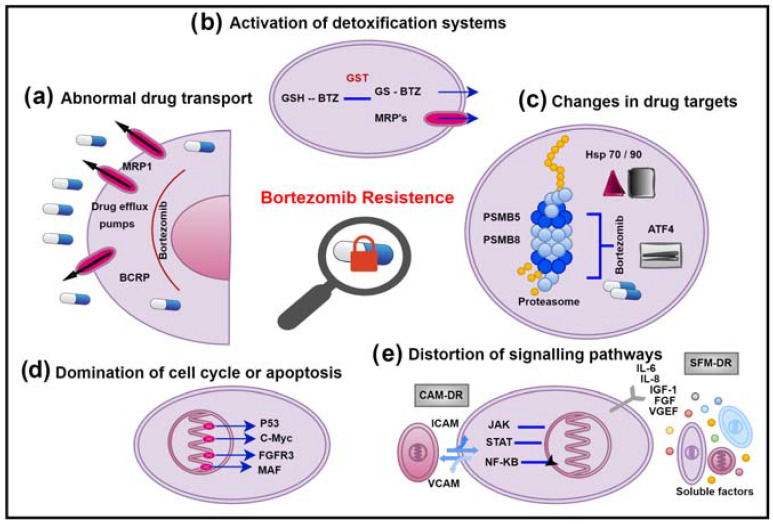
Bortezomib resistance mechanisms in MM. (**a**) Abnormal drug transport. (**b**) Activation of detoxification systems. (**c**) Changes in drug targets. (**d**) Domination of cell cycle or apoptosis. (**e**) Distortion of signalling pathways.

Resistant cells of cancer patients that do not respond to chemotherapy have highly expressed ATP-Binding Cassette (ABC) transporter proteins located in the cytoplasmic region of their membranes [53]. ABC transporters are responsible for transporting drugs and drug metabolites in the organism, working as ATP dependent (Figure 1a). The ABC protein family has at least 48 known members in humans, most of which are drug transporters [54]. A high expression of ABC transporters has been shown to be responsible for MDR [55]. The most studied efflux transporters are ABCB1 (P-glycoprotein), ABCG2 (BCRP), LRP, and MRP1-9. P-glycoprotein (P-gp), the first discovered member of the ABC transporter family, is encoded by the MDR-1 gene [56]. P-gp is the primary drug transporter protein, which binds the drug and carries it against a concentration gradient by ATP hydrolysis [57]. The expression of P-gp in healthy human tissues functions as a natural detoxification mechanism for excreting drugs and other xenobiotics from the body. P-gp is also overexpressed in cancer cells, resulting in a decrease of the intracellular drug concentration by inhibiting the uptake of many structurally different drugs into cells and extruding them from tumour cells [58]. Since most of the routinely used anticancer agents are substrates of P-gp, cancer cells with higher levels of P-gp can develop resistance during adaptation to the treatment [35]. A total of 6% of newly diagnosed MM patients are P-gp positive, while more than 43% are P-gp positive after chemotherapy [28]. In patients with MM, P-gp expression is usually increased after bortezomib, and some studies indicated that Bortezomib is a poor substrate for P-gp [59]. In addition, other studies reported that Bortezomib could reduce P-gp expression in MM cells [60].

Breast Cancer Resistance Protein’s (BCRP) role in normal tissues, similar to P-gp, is preserving the organism as the first line of defence against toxins. It was initially discovered in anthracycline-resistant MCF-7/AdVrp human breast cancer cells [61]. BCRP is prominently expressed in the placenta, small intestine and colon epithelium, liver canalicular membranes, and breast tissue [62]. Increased expression of BCRP was noticed in many drug resistant tumour cell lines [63]. Altered expression in MM cells was associated with drug resistance and poor prognosis [29]. However, BCRP is more expressed in MM stem cells, leading to disease relapse [64].

Lung Resistance-related Protein (LRP) is also called major vault protein (MVP or VAULT1). It was first detected in drug-resistant lung cancer cell lines [65]. Vaults are ribonucleoprotein particles comprising RNA and protein and are found in the cytoplasm as a fraction of the nuclear membrane and nuclear pore complex [66]. Thus, they contribute to drug resistance by transporting substances between the nucleus and cytoplasm. LRP is widely distributed in normal tissues and overexpressed in drug-resistant tumour cells [67]. Overexpression of LRP was reported in leukaemia [68], testicular tumours [69], and breast cancers [70]. In MM, the expression of LRP was observed in patients treated with Melphalan [71] and Bortezomib [29].

The MRP family responsible for MDR includes nine members (MRP-1, MRP-2, MRP-3, MRP-4, MRP-5, MRP-6, MRP-7, MRP-8, MRP-9). MRP-1 is the first member of the family and is expressed in various organ and cell types [72]. The tissue distribution of MRP-1 limits the penetration of certain cytotoxic agents and MRP-1 thus contributes to pharmacological barriers in the body [73]. MRP-1 can carry structurally different kinds of glutathione (GSH) conjugated organic anions [74]. GSH is required for resistance because many studies showed that drug transport occurs only in the presence of reduced GSH [75,76]. MRP-1 has various complex interactions with GSH and GSH, and thus appears to be co-transported with (or cross-stimulates transport of) the drug [77]. Until now, some studies have reported that MRP-1 expression level is high in resistant MM cells [30,35], while others have reported the opposite [60]. MRP-2 is similar to MRP-1 in its ability to confer resistance to a spectrum of anticancer drugs in vitro [31]. MRP-3 expression appears to play a role in compensating for the loss of MRP-2 in liver diseases [32]. A high expression of the MRP-6 gene in resistant tumour cells was found only in cell lines highly expressing the MRP-1 gene [34]. MRP-7 can also carry a large proportion of organic anions in vitro, and it was reported that MRP-7 contributed to various anticancer agents in drug resistance [35,78]. It was suggested that MRP-8 could be a biomarker for predicting the treatment outcomes of AML [36]. Drug resistance caused by ABC transporters is important for MM as in all diseases. Bortezomib is the most potent drug for the treatment of MM, and 2D cell cultures are not sufficient to capture the resistance caused by transporter pumps. The causes of Bortezomib resistance can be revealed by creating a complete model of the bone marrow microenvironment with 3D culture techniques, microfluidic, and organ-on-chip devices. Owing to the delivery of Bortezomib with nanoparticles, fewer side effects and targeted therapy may be possible.

### 2.2. Activation of Detoxification Systems

ABC transporters form a chemo immunity system that dynamically protects our body from the accumulation of foreign chemical agents [79]. While P-gp carries unmodified neutral or positively charged hydrophobic compounds, the members of the MRP family extend the processing time of organic anions and Phase 2 metabolic products. In this sense, it is not a coincidence that GST and P-gp were found to be expressed together in a study [80]. The synergy between detoxification systems and conjugating enzymes composes a very effective system for drug elimination (Figure 1b). Endogenous compounds, lipolic substances’ biosynthesis, and excretion from cells as glutathioneed (GSH), glucoronated, and sulphated xenobiotics are of vital importance in detoxification. These substances are taken up in the cell by oxidation, glutathione, or in conjugation with alternative anionic groups while being extruded from the cell by transporter pumps. Most of the drugs are natural toxins and can also be inactivated by oxidation or conjugation. In Phase 2 reactions, the conjugation with glutathione makes them harmless and water-soluble metabolites. Only conjugation is not sufficient to remove the drug from the cell [81], because such a drug is more hydrophilic. MRP transporters were shown to play a role in detoxification and glutathione-dependent drug resistance [82,83]. 

Glutathione S-transferases (GSTs) conjugate electrophilic and hydrophobic compounds of endogenous or exogenous origin with glutathione. GSTs are a family of enzymes that are generally responsible for Phase 2 detoxification processes, catalysing the conversion to more easily disposable and less toxic metabolites [84]. GSTs comprise various subunits with high polymorphism. Each subunit (22–29 kDa) is a dimeric protein consisting of two catalytically independent functional regions. These functional regions are hydrophilic G-regions that bind the physiological substrate GSH and H-region, which binds the hydrophobic substrates. GSH levels and expression of GST enzymes are increased by the uptake of anti-cancer agent into the tumour cell [85]. Increased GSH/GST levels accelerate the metabolism of many drugs in the treatment of chemotherapy, leading to a lack of drug-targeted effects and resulting in the development of drug resistance [86]. In that case, GST and MRP over expressions are in line with the synergistic effect on high-level resistance to several drugs [35,37]. The results of Zhao et al. with MM patients showed that GSTP1 could be a biomarker for diagnosis and prognosis [38]. In this sense, it is clear that 3D models, microfluidic, and organ-on-chip devices that provide full simulation of the BM microenvironment are needed to prevent bortezomib’s detoxification mechanism and excretion with MDR transporters. In addition, by directing Bortezomib with nanoparticles in a target-specific manner, extra drug use and excretion can be prevented. 

### 2.3. Changes in Drug Targets

The sensitivity of multiple myeloma cells to Bortezomib is based on the fact that malignant B cells depend on protein synthesis and conversion and therefore must rely on the ubiquitin proteasome system (UPS) for processing damaged proteins [87]. Myeloma cells are the most protein-secreting cells of all cell types, and these proteins, if not folded properly, are destroyed in the proteasomes (Figure 1c). Therefore, these cells are under a constant endoplasmic reticulum stress and can easily induce unfolded protein response (UPR) [88]. The efficacy of the proteasome inhibitor Bortezomib is limited by the resistance development in the disease [89]. Studies with MM patient samples and cell lines have shown that Bortezomib resistance is associated with reduced IRE1/XBP1 activity and changes with the activity status of UPR [39]. That resistance is associated with decreased UPS and is also an indicator of disruptions in the mechanisms of autophagy, de-ubiquitation, and chaperone proteins, which allow the cell to overcome this stress [90]. In normal cellular homeostasis, autophagy appears to be a tumour suppressor, while it can direct the tumour cell survival under stress conditions [91]. Autophagy initiates a survival mechanism to eliminate UPS substrates upon proteasome inhibition [92]. In a study with bortezomib-resistant breast cancer cells, it was reported that increased ATF4 expression caused the induction of autophagy [93]. Induction of autophagy via chaperones (Hsp70 and Hsp90) also has an effect on the survival and apoptosis of MM cells [40]. 

The studies on molecular mechanisms underlying Bortezomib resistance have focused on developing BTZ resistant tumour cell line models [94]. BTZ resistant cell line models were mutated in the β5 subunit of the proteasome, and these mutations were clustered at the S1 binding site in the PSMB5 gene [59]. It was observed that different PSMB5 mutations caused different levels of BTZ resistance and continuous mutations occurred due to selective repression in long-term cultures [41]. Of course, only the mutations in the β5 subunit will not be responsible for the entire resistance mechanism. Many studies emphasized that over-expression of the POMP gene plays a role in BTZ resistant cell lines [95]. As known, tumour cells have a potential in directing the immunoproteasome function to get away from immune surveillance [96]. It was shown that the PSMB8 gene responsible for the β5i subunit was mutated in BTZ resistant cell lines [42]. These mutations caused a decrease in PSMB8 expression and chymotrypsin-like activity [97]. This way, BTZ resistant cell lines could gain a high drug resistance phenotype by lowering the immunoproteasome level [98]. siRNA and miRNA technologies can be used to elucidate other proteasome mutations and their functions or other mechanisms for Bortezomib resistance in MM. The use of 3D models, microfluidic, and organ-on-chip devices in combination with siRNA/miRNA technologies will greatly contribute to the examination of the Bortezomib resistance profile at the organism level.

### 2.4. Domination of Cell Cycle or Apoptosis

Cell cycle and apoptosis function in cancer cells are impaired for many different reasons (Figure 1d). Mutations for the activation of oncogenes such as NRAS, KRAS, BRAF, and CCND1 and inhibition of tumour suppressors such as RB1, DIS3, CDKN2A, and CDKN2C are involved in the development of MM [99]. P-53, known as the guardian of the genome, is a protein that has excessive mutations in cancer patients and fulfils this role by mediating the degradation of numerous cell cycle regulators and apoptotic factors (Bcl-2, p21, p27, c-Myc, cyclin A, B, D, E). When p53 mutation occurs in MM cells, the relevant signalling pathways and targets were shown to cause the development of anti-apoptosis and drug resistance [43,46]. The overexpression of c-Myc on chromosome 8q24 is also associated with disease aggression and Bortezomib resistance [44]. MM cells with MAF overexpression were resistant to Bortezomib by inhibiting apoptosis [45]. All of these contribute to oncogenesis by promoting MM progression and drug resistance.

Apoptosis is an energy-dependent programmed cell death, regulated by the organism. This process plays a critical role in the maintenance of tissue homeostasis as well as the destruction of damaged or potentially dangerous cells. Chemotherapy can substantially kill tumour cells with apoptosis, while inhibition of apoptosis can make tumour cells become resistant to chemotherapy [100]. Suppressing apoptosis provides an advantage to the cancer cell by reducing cell forfeit [101]. MM cells induce apoptosis by changing FAS, TNF-associated ligands, or Bcl-2/Bax ratio [46]. Changes in Bcl-2 and Bax regulation were observed in MM cells following Bortezomib treatment [102]. Apoptosis-suppressed MM cells increase the regulation of antiapoptotic factors (Bcl-xL, Mcl-1, Bcl-2), upregulate apoptosis inhibitors, and acquire resistance to FAS, TNF ligands that induce apoptosis [47]. TNF-α and FasL family member TRAIL/Apo2L reversed Bortezomib resistance in MM cells [103]. MM cells express high levels of PD-L1, which helps them evade immune cells. Increased PD-L1 expression in MM cells stimulated with IFN-γ and TLR ligands escapes from cytotoxic T lymphocytes by inhibition of MyD88/TRAF6 and MEK/ERK/STAT1 [104]. The resistant tumour cells get rid of drug-induced apoptosis by excreting the drug from the cell, and ABC transporters cause multi-drug resistance in tumour cells not only by drug excretion but also by apoptosis and cell cycle signalling pathways [105]. Furthermore, glutathione conjugates of many anti-cancer drugs regulate the stress-activated apoptosis pathway through GST isoenzymes [106]. 

Apart from these, it was noticed that some miRNAs targeting genes that regulate cell cycle, apoptosis, survival and cell growth in MM are dysregulated [107]. For example, miR-106b-25 cluster, miR-181a, miR-181b, and miR-32 together regulate p-53 [108]. The miR-17-92 cluster regulates Bcl-2 [109], miR-29b Mcl-1 [110], miR-21 STAT3 [111], and miR-125b BLIMP1 [112]. Furthermore, Neri et al. identified an MM miRNA signature that was critical in the development of resistance to Bortezomib [48]. The genetic mechanisms responsible for the development of Bortezomib resistance in multiple myeloma can be complex [113]. Therefore, 3D modelling of the factors that push MM cells to apoptosis and resistance to bortezomib, both in terms of genetics and the tumour microenvironment, might be a solution for re-sensitizing myeloma cells. Gene silencing and personalized treatment options will be possible with microfluidic and organ-on-chip devices that can be used for this purpose. Moreover, with the targeting ability of nanoparticles, specifically MM cells will be able to undergo apoptosis.

### 2.5. Distortion of Signalling Pathways

Inhibiting proteasomes with Bortezomib disrupts various cell signalling pathways, leading to apoptosis, cell cycle arrest, and supressing angiogenesis (Figure 1e). Cancer cells can prevent drug-induced apoptosis by activating survival factors. The interaction of myeloma cells between bone marrow (BM) stromal cells and extracellular matrix (ECM) proteins is vital for ensuring the release of growth factors and cytokines. Bortezomib prevents the binding of myeloma cells to ECM proteins and BM stromal cells. The proliferation of MM cells is triggered by cytokines such as IL-6, IL-21, IGF-1, VEGF, TNF-α, SDF-1α, and the RAF/MEK/MAPK signalling cascade in the BM microenvironment [114]. NF-kB activity in myeloma cells is important for maintaining the interaction with BM stromal cells, because this factor regulates the expression of IL-6, VEGF, and IGF-1, which provides the survival, development, and chemoresistance of myeloma cells around BM [49]. Subsequently, JAK/STAT3 and PI3K/AKT signal cascades take place. NF-kB allows the expression of genes that protect cells from drug-induced apoptosis, thereby reducing the effectiveness of chemotherapy [50]. While Bortezomib performs apoptotic function by inhibiting the canonical pathway of NF-kB, it induces the non-canonical pathway that makes myeloma cells less susceptible to Bortezomib at the same time [115]. Thus, myeloma cells could develop a bortezomib-resistant NF-kB phenotype [116]. Inhibition of NF-kB activation might cause DNA damage via the atypical pathway in myeloma cells and result in the actuation of multiple survival mechanisms. 

BM microenvironment mediated drug resistance is defined by soluble factor (SFM-DR) and cell adhesion (CAM-DR) drug resistance mechanisms [117]. MM precursor cells with a high expression of adhesion molecules are drug resistant and selected with the contribution of CAM-DR during treatment process [51]. SFM-DR can be best described by IL-6 affinity, because IL-6 secretion leads to Bortezomib resistance in myeloma cells [52]. Likewise, myeloma cells showed Bortezomib resistance by IL-8 released from BM stromal cells [118]. In addition, MARCKS is a protein that plays an important role in cell adhesion, spreads invasion, and has recently been implicated in metastasis [119]. The ability of exosomes to deliver various molecules might affect Bortezomib resistance. Indeed, it was reported that BMSC-derived exosomes could inhibit MM cell death [120]. It was also found that increased RARα-2 expression contributed to drug resistance in MM CSCs [121]. With the contributions of 3D culturing, microfluidic and organ-on-chip devices that recapitulate the disrupted signalling pathways and tumour microenvironment, we can understand how the processes lead to MM Bortezomib resistance function. Thus, personalized and bedside treatment will be possible, and new generation drugs and different advancing technologies will be realized.

## 3. Emerging Technologies

Emerging technologies provide valuable platforms to overcome Bortezomib resistance in MM. Nanoparticles, 2D and 3D cell models, microfluidic and organ-on-chip devices are covered in this review (Table 3). The related technologies assist us in unravelling the complex mechanisms of MM such as tumour microenvironment, invasion, metastasis, apoptosis, drug delivery, and drug resistance.

### 3.1. Nanoparticles

**Figure 2:**
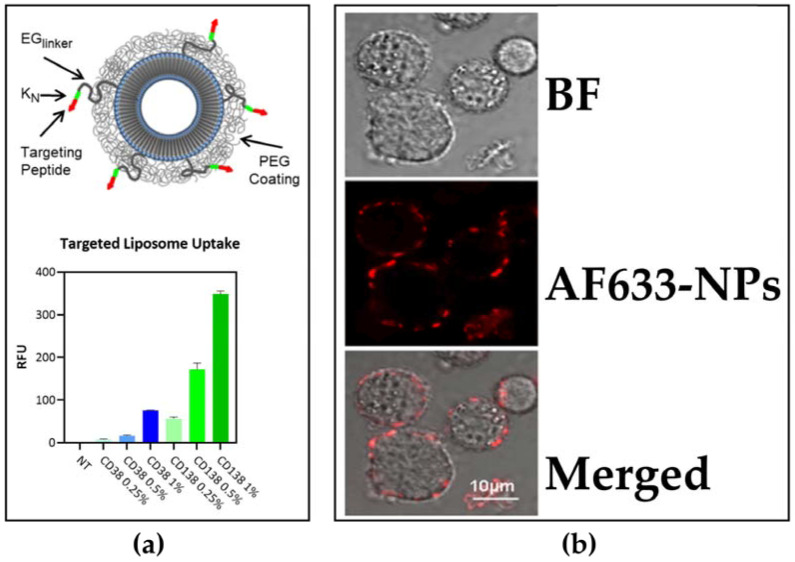
(**a**) CD38 or CD138 peptide-conjugated liposomal nanoparticle and its uptake by MM cells [123], Copyright 2020, Journal of Hematology and Oncology; (**b**) fluorescence reading of MM1s cells after 2-h exposure to AF633 anti-CD38 chitosan nanoparticles. BF (Bright field); AF633 (Red) [124], Copyright 2018, Journal of Controlled Release.

By overcoming the molecular complexity of tumour cells, smart drug methods are used to better modulate intercellular signalling and to attract immune cells. They are indispensable for treatment and utilization procedures, and can be directed to the bone marrow where MM cells originate, maximizing the therapeutic effect and thus minimizing the side effects of chemotherapeutics [145,146]. Most of the chemotherapeutics used in cancer are eliminated by detoxification systems in the body. Drug delivery systems enable the chemotherapeutics used to reach the desired area in the body at the desired dose and to treat them with the least side effects [145]. Nanoparticles are 1–1000 nm in size, and they are biocompatible, biodegradable, and can be loaded with more than one therapeutic agent. Drug delivery with nanoparticles can overcome the drawbacks of combined drug therapy, multi-drug resistance, low drug efficacy, and limited drug regimen [147]]. Nanoparticles are the encapsulation of a drug or a new chemical agent through micelles, liposomes, dendrimers, nanospheres, etc. [146]. Liposomes have more success because of their higher biocompatibility [147]. For instance, ROCK inhibitor and Bortezomib loaded liposomes were more effective on MM cells and caused less side effects in a study targeting the bone marrow microenvironment [122]. Peptide-conjugated liposomal nanoparticles targeting MM via CD38 and CD138 receptors were considered as more promising (Figure 2a) [123]. In another CD38-targeted study, it was reported that chitosan Bortezomib nanoparticles increased proteasome inhibition as a result of endocytosis uptake (Figure 2b) [124].

**Figure 3:**
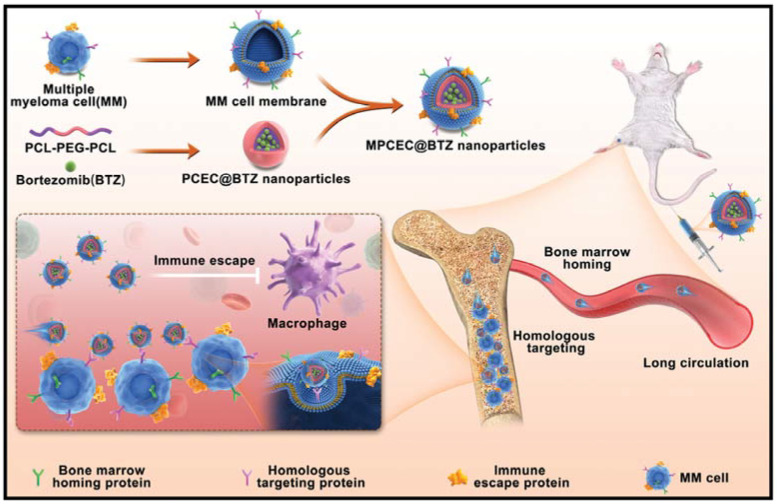
Targeting the tumour cells of nanoparticles loaded with Bortezomib and coated with MM cell membrane [125], Copyright 2022, Advanced Materials.

Nanoparticles can be prepared using polymer, non-polymer and lipid-based materials. Polymer-based ones include dendrimers, nanoparticles, micelles, and nanogels, while the most notable ones among non-polymer-based ones are silica-based, metallic nanoparticles, and nanotubes [145]. The nanoparticles obtained by covering Bortezomib-loaded polymers with myeloma cell membrane can escape from phagocytosis and easily enter the BM microenvironment because they resemble the MM cells (Figure 3) [125]. PEGylated dendrimer Bortezomib-prodrug was also shown to be important in the treatment of solid tumours in vivo [126]. Hyaluronic acid shell and disulphide-crosslinked core micelles with Bortezomib distribution exhibited growth suppression in mice [127]. The physical and chemical properties, size, and shape of nanoparticles can vary according to the material used in their preparation. Therefore, the particle must be well designed in order to display the desired effect. For example, Bortezomib mesoporous silica vehicle targeted to the folic acid receptor on MM cells was very successful in apoptosis [128]. In another study on targeting folic acid, Bortezomib loaded on gold nanoparticles maintained their functional activity [129]. The drug and polymer combination makes the drug more soluble in water [148]. The success of drug-polymer complexes in therapy could be seen in Doxil, which was approved by the FDA in 1995 [149]. MM cells were treated with polymer-coated 5-Aza-2′-deoxycytidine and Bortezomib nanoparticles to enhance apoptosis [130].

Targeting the nanoparticles to cancer cells is essential with the use of active and passive approaches. The enhanced permeability and retention (EPR) effects in the passive targeting make nanoparticles easily arrive and accumulate in the tumour region, where vascularization is higher [145]. In active targeting, the nanoparticle surface is coated with targeting fragments such as proteins, peptides, nucleic acids, antibodies, or small molecules that recognize and bind cancer or endothelial cell receptors and stimulate endocytosis [146]. Although nanoparticles should be target-specific, they should not damage other tissues and should be easily eliminated from the body after performing their role. Mechanisms that can be triggered by pH, temperature, magnetic field, ultrasound, or light are designed for the controlled release of nanodrugs. Nanoparticles have been investigated frequently in the treatment of bortezomib-resistant MM. However, these studies are still in the experimental stage and none of them have been clinically tested and turned into commercial products. Apart from the treatment option, nanoparticles can also be used for diagnostic purposes. Theranostics generated with imaging or positioning agents, which are engaged to nanoparticles, enable drug delivery, release, and efficacy [150]. These platforms also allow a non-invasive cancer diagnosis. 

### 3.2. 2D/3D Culture Systems

**Figure 4:**
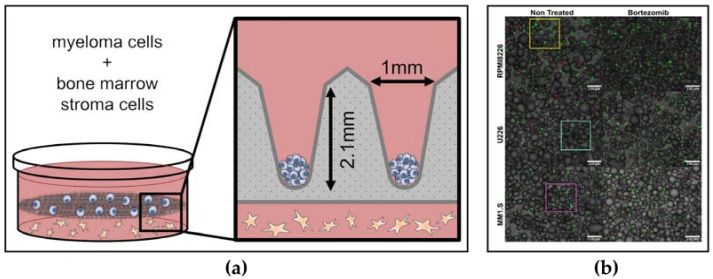
(**a**) MM cells and BM stroma cells in 3D coculture [131], Copyright 2022, Journal of Cancer Research and Clinical Oncology. (**b**) Live (green) and Dead (red) states on 10% AA microgels of three MM cell lines untreated or exposed to BTZ 4 nM for 72 h [133], Copyright 2022, Biomaterials Advances.

As a conventional approach, tumour migration and invasion can be investigated in 2D cell culture systems. However, developing 3D techniques is more successful in mimicking the tumour microenvironment. The conditions for proliferation, differentiation, and stimulation are more similar to the ones an in vivo environment in 3D cell cultures. For example, the 3D coculture of primary MM cells on a conical agarose microwell array exhibited a more robust proliferation than 2D and liquid overlay cultures (Figure 4a) [131]. Furthermore, Bortezomib and Auranofin caused more cytotoxicity in the 3D culture [131]. In a human bone marrow-like microenvironment produced by 3D bioprinting, MM cells were able to show better responses to Bortezomib [132]. Accordingly, scaffolds can be produced organically or synthetically from a basis where tumour cells can unite and mature. Scaffolds provide the cancer cell with ECM and TME task required for adhesion, signalling, and proliferation. Therefore, the type, surface area, and pore structure of the material used in the research are important. Spheroids are frequently used in cancer research to elucidate cell-cell or cell-ECM relationships and to interpret signalling pathways and gene expressions [151]. In a 3D culture platform designed with microspheres and microgels, MM cells were able to proliferate and also represented the resistance to Bortezomib (Figure 4b) and Dexamethasone [133]. Tumour-associated macrophages (TAMs) accumulate in the TME, reducing the immune response of T cells. In a 3D culture that mimics the cancer immune environment, the regulation of T-cell immunotherapy by TAM could be investigated [134]. Additionally, CAR-T cells that can be cocultured with spheroids can better recognize cancerous cells and contribute to immunotherapy. However, 3D techniques cannot provide the full picture of inter-tissue contact, concentration gradients, and mechanical properties in organs [152]. Due to the short lifespan of primary cells taken from patients, clinical trials are challenging but not impossible. By integrating 3D culturing technology with microfluidic and organ-on-chip devices, the processes that lead MM cells to Bortezomib resistance can be masticated in a more economical fashion within a short time.

### 3.3. Microfluidic Systems

Microfluidic devices offer attractive conditions such as low Reynolds numbers, reduced sample volume, the ability to perform multiple assays on a single chip, fast reaction time, control of conditions, portability, and point-of-care application, and could be utilized in laboratory tests of blood, urine, or any fluid [153]. The chemical properties of the reservoirs providing a suitable platform for biological samples are vital for biomedical applications of microfluidic systems. Polydimethylsiloxane (PDMS) and polymethyl methacrylate (PMMA) are biocompatible materials most often used in microfluidic devices as host materials. Transparency is another parameter for microscopy analysis during biological activations. The internal environment must be under suitable conditions for the survival of cells or biological samples. As transparency permits, oxygen, carbon dioxide, or other chemical compounds could be detected using optical methods. While it is challenging to use biocompatible materials, the difficulties in their use have been overcome by developing various fabrication techniques such as moulding, 3D printing, nanofabrication, and etching [154]. Microfluidic cell culturing chambers developed with soft lithography, which is a subbranch of moulding techniques, have been used in haematological cancer studies [155], drug delivery [156], and treatment of bortezomib-resistant MM [157]. In this regard, microfluidic devices have been developed and operated under laminar flow conditions, thereby providing suitable protective conditions for biological systems. Microfluidics allows Polymerase Chain Reaction (PCR), electrophoresis, and hybridization techniques to be performed on a single chip. As a result of the sensitivity of microfluidic technology, small amounts of DNA, RNA, and Circulating Tumour Cells (CTC) biomarkers in the blood could be used in the diagnosis of cancer. 

**Figure 5:**
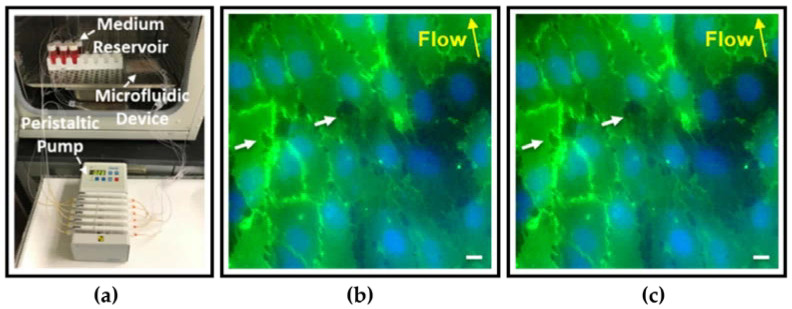
(**a**) A microfluidic device that mimics the circulation of MM cells through the BM sinusoidal endothelium [138], Copyright 2022, Scientific Reports. (**b**,**c**) Endothelial layer formed after 30 h of culture CD31 (green) and DAPI (blue). White arrows show pores, yellow arrows show the direction of flow adopted from [138], Copyright 2022, Scientific Reports.

Microfluidics assists in appreciating tumour intravasation, extravasation, and angiogenesis by creating a physically and chemically controllable biological environment [153]. It is known that tumour cells can easily migrate from capillaries to other regions through invasion and cause metastasis in these regions. Microfluidic systems can manipulate the fluid flow physics of the circulatory system. After tumour cells enter the bloodstream, they can be physically damaged or exposed to immune attacks, as nearby vessels have varying rates of blood viscosity, and this shear stress is involved in various cancer-related events such as extravasation and metastasis [152]. Traffic and metastasis of MM cells can be examined with a microfluidic device that recapitulates bone marrows’ stroma, sinusoidal endothelium, and circulation (Figure 5) [138]. In addition, a mechanical property-based microfluidic platform is useful to capture clonal plasma cells [139]. In clinical trials, a microfluidic device that enables the precise detection of cytogenetic changes in plasma cells could be developed [142]. The epithelial-mesenchymal transition (EMT) process can be mimicked by creating concentration gradients of various growth factors. The small size of the microfluidic systems contributes to the fruitfulness of drug screening, and makes it possible to test chip-sized drug candidates, and to perform immunotherapy and genetic therapy applications [158]. Thanks to microfluidic technology, we can better understand the processes of invasion, metastasis, and drug resistance by simulating the MM bone marrow tumour microenvironment. However, clinical trials regarding the microfluidic technology are few and need to be expanded. Furthermore, droplet microfluidics, involving the confinement and capillary forces of two immiscible liquids passing through a channel, makes a difference in encapsulating various biological molecules, drug screening, and drug resistance studies [140,159].

### 3.4. Organ on a Chip

**Figure 6:**
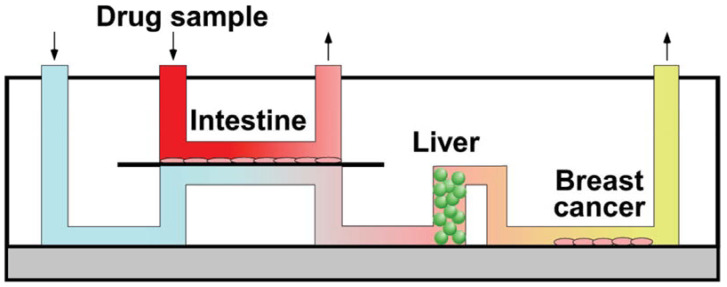
Multiorgan-on-chip to mimic metastasis. Caco-2 cells for the intestine, HepG2 cells for the liver, and MCF-7 cells for breast cancer were cultured [165], Copyright 2010, American Chemical Society.

A new era in cancer research has started mimicking the process in organs with the use of miniature devices. This technology can be used for both single cell research and intercellular communication studies by mimicking the TME environment, because it provides a recapitulation of cancerous tissues and organs in small scale, particularly for cases where animal models are not sufficient (Figure 6). The tumour mass contains not only cancer cells but also ECM elements, stromal cells, immune cells, and capillaries. Organ-on-a-chip devices can offer many features such as concentration gradient and shear force in microfluidics with cell/tissue/organ interactions in living organisms [24]. In its simplest form, the activity of cells against drugs can be observed with this technique where a single type of cell is seeded in a channel on the chip and is prevented from another media [160]. Flow characteristics, nutrients, oxygen, mechanics, and shear forces in this technology should be well tuned for a complete recapitulation of the processes in the organism. In light of these efforts, it is possible to generate organs and tissues-on-chip platforms, where organs and tissues can be modelled. In this way, gene expressions, signalling pathways, apoptosis, and tumour development processes that cause drug resistance can be preclinically examined. Accordingly, a small-scale imitation of organs such as heart, lung, liver, kidney, and tissues such as skin, gut, and vessels could be fabricated [161,162]. In the following stages, body-on-a-chip devices can be achieved by combining all these organ and tissue models [163]. In this manner, a model of any disease could be easily contrived, and new drug candidates or drug-resistance researches can be performed on these models without the need for animal experiments [164]. 

**Figure 7:**
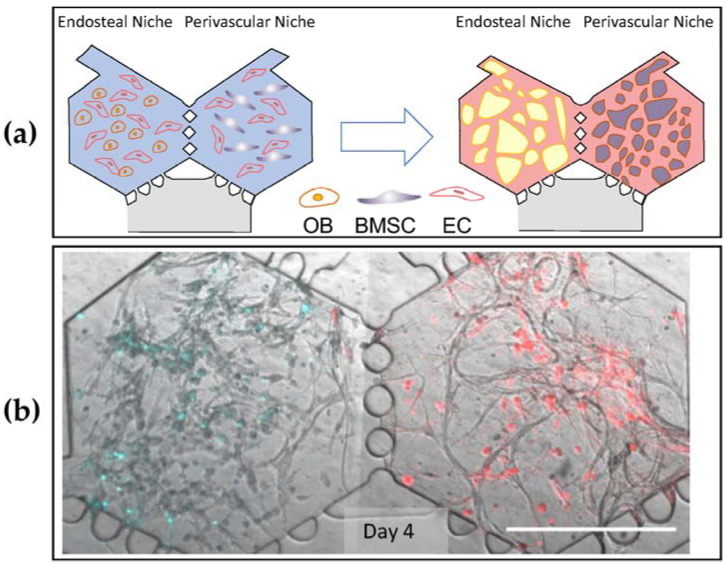
(**a**) Bone-on-chip schematic representation. Cord blood-derived endothelial cells (EC) seeded with either hFOB (OB, Endosteal Niche) or BMSC (Perivascular Niche) form microvascular networks in a period of 4–7 days [144], Copyright 2022, Biomaterials. (**b**) BMoaC cultured with CellTracker™ Violet hFOB and Deep Red BMSC demonstrated stromal separation by niche [144], Copyright 2022, Biomaterials.

To date, few bone-on-chip studies have been conducted, but more research efforts and clinical trials are needed for specific bone marrow diseases such as MM [166,167,168]. Because bone is a highly vascularized tissue, it is important to mimic the osteogenic microenvironment. The model closest to the in vivo bone marrow microenvironment was able to demonstrate the maintenance and differentiation of CD34^+^ hematopoietic stem/progenitor cells, outgrowth of neutrophils (CD66b^+^), and niche-specific responses to doxorubicin and granulocyte-colony stimulating factor (Figure 7) [144]. The chip developed in another study supports the differentiation and maturation of multiple blood cell lineages while recapitulating many clinically relevant features of BM pathophysiology in response to drugs, radiation, and genetic mutation [169]. However, summarizing only malignant B cells and accompanying stromal cells will be insufficient for MM disease. In order to understand the invasion and metastasis processes, multiorgan-on-chip devices to be used in preclinical trials are needed. 

## 4. Future Research Directions

MM is still a deadly disease. Even though Bortezomib is widely used in the treatment of MM and is an effective chemotherapeutic, its drug resistance constitutes a significant issue. It is very clear that nanoparticles will help to prevent Bortezomib resistance and the side effects. Besides, integration of Bortezomib nanoparticles with microfluidic systems for the treatment of MM will be beneficial in overcoming resilience. In fact, microfluidic systems should be designed for the use of these nanoparticles in the clinic as an MRI device. 

Bortezomib is usually given to patients intravenously, which causes pain in patients. Even if oral Ixazomib is another alternative, there are cases where only Bortezomib should be used in the clinic. The quality of life of patients who have to cope with the pain of peripheral neuropathy is already decreased. For this reason, it can be ensured that Bortezomib treatment, which is currently applied in the clinic, can be less painless. New technological approaches such as microneedles allow for more painless intravenous delivery of bortezomib, and metabolic degradation of Bortezomib and resistance to Bortezomib by overdose can be thus prevented [170,171]. With this approach, we can offer patients a more comfortable life by avoiding the side effects of Bortezomib. 

Although it has been attended in the literature to understand the molecular mechanisms that cause Bortezomib resistance in MM, it cannot be claimed that the resistance and responsive mechanisms have been fully assessed. It seems unlikely that we can both understand this resistance and develop new therapeutic approaches in conventional culturing methods. For this reason, processes such as proliferation, apoptosis, metastasis, and angiogenesis in MM should be carried out with new generation culture methods and state-of-the-art miniaturized devices. Thanks to the 3D cell cultures, MM cells are most closely represented in their natural environment. However, studies so far have tried to summarize MM harvesting with only a few cell groups. In addition, it is necessary to improve the 3D systems, which include stem cells, immune, and bone cells, that summarize the complete bone marrow. In this way, it will be possible to examine the molecular mechanisms and re-sensitization pathways that trigger Bortezomib resistance. Combining gene silencing systems such as siRNA/miRNA with 3D culturing and microfluidic systems can simulate step-by-step genetic mechanisms, leading to Bortezomib resistance. It is a disadvantage that primary 3D cultures survive for a few days, and the observation of processes such as cell interactions, invasion, and metastasis within such a short time is also limited [172]. For this reason, personalized treatment approaches do not seem very possible in the near future.

Although the use of microfluidic devices in MM has shown success in recapitulating the microenvironment, it is undeniable that more research is needed. New generation microfluidic systems to be developed should be able to mimic the behaviour of MM cells and contact the bone marrow microenvironment. In addition, these devices should be easy to fabricate and cost effective. Moreover, MM is not a disease that only affects bones and related tissues. The immune cells and blood cells of the individual are also affected, and in fact, all tissues are at risk of metastasis through the blood. Although not specific to MM, several successful bone-on-chip studies have been conducted [144]. However, it cannot be stated that the complete bone marrow was summarized in these studies, and all cells belonging to the bone tissue were used. Since MM is a disease that can affect the whole body, multiorgan-on-chip and body-on-chip studies need to be performed. Finally, microfluidic and organ-on-chip devices need to be used in the clinic. Developing point-of-care systems for cell isolation from patients, accelerating diagnosis with cancer biomarkers and predicting metastasis processes will be vital. In order to achieve all these goals, it is necessary to improve academic research efforts, as well as joint collaborations with health industry. This way, cancer will become less deadly.

## Figures and Tables

**Table 1:** The effects of Bortezomib on different tumors.

Cancer Type	Effect	Ref.
Adult T-cell leukemia/Cutaneous T-cell lymphoma	Inactivation of nfκb pathway and up-regulation of NOXA	[11]
Breast cancer	Activation of caspase-3 in p53-null breast cancer cells	[12]
Cervical cancer	Increased expression of caspase-3, PARP, and increased the level of ER stress-associated and autophagy-related proteins	[13]
Colorectal carcinoma	Prevention of NF-κb signaling	[14]
Esophageal squamous cell carcinomas	TRAIL-induced apoptosis and increased Association of caspase-8 and the Fas-associated death domain	[15]
Head and neck squamous cell carcinomas	Inhibition of NF-κb and AP-1 activities	[16]
Melanoma	Activation of ER-stress and mitochondrial-dysregulation associated pathways	[17]
Neuroblastoma	Induction of eif2α signalling and ATF-4 dependent ER stress	[18]
Non-small lung cancer	Up-regulation of p21(waf1) and p53, and down-regulation of bcl-2 via the JNK/c-Jun/AP-1 signaling	[19]
Pancreatic cancer	Repression in Bcl-2 and an Increase in Bax and p53	[20]
Prostate cancer	Inhibition of HIF-1α and suppression of PI3K/Akt/mtor and MAPK pathways	[21]
Renal cell carcinoma	Increase in caspase-8 activity	[22]

**Table 2:** Summary table of Bortezomib resistance mechanisms in MM.

Resistance Mechanism	Main Factors	Contribution	Ref.
**Abnormal drug transport**	P-gp, BCRP, LRP, MRP1-9	Increases Bortezomib excretion	[28,29,30,31,32,33,34,35,36]
**Activation of detoxification systems**	GSH/GST levels	Increases Bortezomib excretion	[37,38]
**Changes in drug targets**	Unfolded Protein Response, Autophagy, PSMB5 and PSMB8 mutations	Prevents Bortezomib binding to proteasome by interrupting the UPS	[39,40,41,42]
**Domination of cell cycle or apoptosis**	P-53, c-Myc, MAF, Bcl-2/Bax ratio, anti-apoptotic factors, miRNAs	Regulates cell survival and death	[43,44,45,46,47,48]
**Distortion of signaling pathways**	NF-kB, JAK/STAT3, PI3K/AKT, SFM-DR, CAM-DR	Maintains interaction with BM microenvironment	[49,50,51,52]

**Table 3:** Emerging technologies used in MM disease.

Emerging Technology	Method	Anticancer Agent	Purpose	Ref.
**Nanoparticles**	Liposome	Bortezomib	Drug delivery	[122,123]
	Chitosan	Bortezomib	Drug delivery	[124]
	Poly(ε-caprolactone)-poly(ethylene glycol)-poly(ε-caprolactone) with the MM cell membrane	Bortezomib	Drug delivery	[125]
	PEGylated dendrimer	Bortezomib	Drug delivery	[126]
	Hyaluronic acid shell and disulfide-crosslinked core micelles	Bortezomib	Drug delivery	[127]
	Mesoporous silica	Bortezomib	Drug delivery	[128]
	Gold nanoparticle	Bortezomib	Drug delivery	[129]
	Polyethylene glycol and polycaprolactone	5-Aza-2ʹ-deoxycytidine Bortezomib	Drug delivery	[130]
**3D culture systems**	Conical agarose microwell array	Bortezomib and Auranofin	3D high-throughput co-culture system	[131]
	Coaxial extrusion bioprinting	Bortezomib Tocilizumab	3D-Bioprinted multiple myeloma model	[132]
	Microspheres Microgels	Bortezomib Dexamethasone	Dynamic 3D multiple myeloma culture	[133]
	TAM modulation of cancer immunotherapy	-	Ex vivo 3D TME-mimicry culture	[134]
	3D myeloma coculture with bone cell/cancer cell	-	Investigation of MM cells osteogenesis, angiogenesis, tumor growth, and drug response	[135]
	2D/3D coculture	Withaferin A	Cytotoxicity	[136]
	2D/Hydrogel based 3D ex vivo co-culture system	Pomalidomide Lenalidomide Thalidomide Bortezomib Carfilzomib Doxorubicin Dexamethasone Melphalan	MM pathogenesis and drug resistance in the BM niche	[137]
**Microfluidic systems**	Traffic and metastasis of MM cells	-	Mimic bone marrows’ stroma, sinusoidal endothelium and circulation	[138]
	Capture clonal plasma cells	-	Micropillar-integrated microfluidic device	[139]
	Micromanipulation and encapsulation using a droplet-based microfluidic device	Bortezomib Lenalidomide	Ex vivo platform of primary multiple myeloma cells for drug screening	[140]
	Thermoplastic PDMS microfluidic devices	Bortezomib Carfilzomib	The importance of material selection in microfluidic device design, for drug cytotoxicity	[141]
	Enrichment of plasma cells by MF-CD45-TACs (microfluidic–CD45 depletion–tetrameric antibody complexes)	-	Detection of cytogenetic abnormalities in MM patients	[142]
	Propagation of primary CD138^+^ MM cells in microfluidic-cis-culture (MicroC^3^) to simulate patients’ own tumor microenvironment	Bortezomib	Chemosensitivity and resistance assay	[143]
**Organ-on-chip**	Organ-on-a-chip model of vascularized human bone marrow niches	Doxorubicin	3D in vitro model of human bone marrow function and drug response	[144]

## Data Availability

Not applicable.

## References

[1] Méndez-Ferrer S., Bonnet D., Steensma D.P., Hasserjian R.P., Ghobrial I.M., Gribben J.G., Andreeff M., Krause D.S. (2020). Bone marrow niches in haematological malignancies. Nat. Rev. Cancer.

[2] Mateos M.V., Ludwig H., Bazarbachi A., Beksac M., Bladé J., Boccadoro M., Cavo M., Delforge M., Dimopoulos M.A., Facon T. (2019). Insights on Multiple Myeloma Treatment Strategies. HemaSphere.

[3] Institute N.C. Surveillance, Epidemiology, and End Results Program. https://seer.cancer.gov/.

[4] Ito S. (2020). Proteasome Inhibitors for the Treatment of Multiple Myeloma. Cancers.

[5] Lipchick B.C., Fink E.E., Nikiforov M.A. (2016). Oxidative stress and proteasome inhibitors in multiple myeloma. Pharmacol. Res..

[6] Auner H.W., Cenci S. (2015). Recent advances and future directions in targeting the secretory apparatus in multiple myeloma. Br. J. Haematol..

[7] Siegel D.S., Martin T., Wang M., Vij R., Jakubowiak A.J., Lonial S., Trudel S., Kukreti V., Bahlis N., Alsina M. (2012). A phase 2 study of single-agent carfilzomib (PX-171-003-A1) in patients with relapsed and refractory multiple myeloma. Blood.

[8] Dimopoulos M.A., Schjesvold F., Doronin V., Vinogradova O., Quach H., Leleu X., Montes Y.G., Ramasamy K., Pompa A., Levin M.-D. (2022). Oral ixazomib-dexamethasone vs. oral pomalidomide-dexamethasone for lenalidomide-refractory, proteasome inhibitor-exposed multiple myeloma: A randomized Phase 2 trial. Blood Cancer J..

[9] Adams J., Palombella V.J., Sausville E.A., Johnson J., Destree A., Lazarus D.D., Maas J., Pien C.S., Prakash S., Elliott P.J. (1999). Proteasome inhibitors: A novel class of potent and effective antitumor agents. Cancer Res..

[10] Boccadoro M., Morgan G., Cavenagh J. (2005). Preclinical evaluation of the proteasome inhibitor bortezomib in cancer therapy. Cancer Cell Int..

[11] Ri M., Iida S., Ishida T., Ito A., Yano H., Inagaki A., Ding J., Kusumoto S., Komatsu H., Utsunomiya A. (2009). Bortezomib-induced apoptosis in mature T-cell lymphoma cells partially depends on upregulation of Noxa and functional repression of Mcl-1. Cancer Sci..

[12] Yerlikaya A., Okur E., Ulukaya E. (2012). The p53-independent induction of apoptosis in breast cancer cells in response to proteasome inhibitor bortezomib. Tumor Biol..

[13] Zhang Y., Bai C., Lu D., Wu X., Gao L., Zhang W. (2016). Endoplasmic reticulum stress and autophagy participate in apoptosis induced by bortezomib in cervical cancer cells. Biotechnol. Lett..

[14] Voutsadakis I.A., Patrikidou A., Tsapakidis K., Karagiannaki A., Hatzidaki E., Stathakis N.E., Papandreou C.N. (2010). Additive inhibition of colorectal cancer cell lines by aspirin and bortezomib. Int. J. Colorectal. Dis..

[15] Seki N., Toh U., Sayers T.J., Fujii T., Miyagi M., Akagi Y., Kusukawa J., Kage M., Shirouzu K., Yamana H. (2010). Bortezomib sensitizes human esophageal squamous cell carcinoma cells to TRAIL-mediated apoptosis via activation of both extrinsic and intrinsic apoptosis pathways. Mol. Cancer Ther..

[16] Chen Z., Ricker J.L., Malhotra P.S., Nottingham L., Bagain L., Lee T.L., Yeh N.T., Van Waes C. (2008). Differential bortezomib sensitivity in head and neck cancer lines corresponds to proteasome, nuclear factor-κB and activator protein-1 related mechanisms. Mol. Cancer Ther..

[17] Selimovic D., Porzig B.B.O.W., El-Khattouti A., Badura H.E., Ahmad M., Ghanjati F., Santourlidis S., Haikel Y., Hassan M. (2013). Bortezomib/proteasome inhibitor triggers both apoptosis and autophagy-dependent pathways in melanoma cells. Cell. Signal..

[18] Armstrong J.L., Flockhart R., Veal G.J., Lovat P.E., Redfern C.P. (2010). Regulation of endoplasmic reticulum stress-induced cell death by ATF4 in neuroectodermal tumor cells. J. Biol. Chem..

[19] Yang Y., Ikezoe T., Saito T., Kobayashi M., Koeffler H.P., Taguchi H. (2004). Proteasome inhibitor PS-341 induces growth arrest and apoptosis of non-small cell lung cancer cells via the JNK/c-Jun/AP-1 signaling. Cancer Sci..

[20] Awasthi N., Schwarz M.A., Schwarz R.E. (2010). Combination effects of bortezomib with gemcitabine and EMAP II in experimental pancreatic cancer. Cancer Biol. Ther..

[21] Befani C.D., Vlachostergios P.J., Hatzidaki E., Patrikidou A., Bonanou S., Simos G., Papandreou C.N., Liakos P. (2012). Bortezomib represses HIF-1α protein expression and nuclear accumulation by inhibiting both PI3K/Akt/TOR and MAPK pathways in prostate cancer cells. J. Mol. Med..

[22] Brooks A.D., Jacobsen K.M., Li W., Shanker A., Sayers T.J. (2010). Bortezomib sensitizes human renal cell carcinomas to TRAIL apoptosis through increased activation of caspase-8 in the death-inducing signaling complex. Mol. Cancer Res..

[23] Shen C., Ferro E.G., Xu H., Kramer D.B., Patell R., Kazi D.S. (2021). Underperformance of Contemporary Phase III Oncology Trials and Strategies for Improvement. J. Natl. Compr. Cancer Netw..

[24] Zuchowska A., Skorupska S. (2022). Multi-organ-on-chip approach in cancer research. Organs-on-a-Chip.

[25] Schmitt S.M., Deshmukh R.R., Dou Q.P., Dou Q.P. (2014). Proteasome Inhibitors and Lessons Learned from Their Mechanisms of Action and Resistance in Human Cancer. Resistance to Proteasome Inhibitors in Cancer: Molecular Mechanisms and Strategies to Overcome Resistance.

[26] Maiso P., Huynh D., Moschetta M., Sacco A., Aljawai Y., Mishima Y., Asara J.M., Roccaro A.M., Kimmelman A.C., Ghobrial I.M. (2015). Metabolic signature identifies novel targets for drug resistance in multiple myeloma. Cancer Res..

[27] Wu Q., Yang Z., Nie Y., Shi Y., Fan D. (2014). Multi-drug resistance in cancer chemotherapeutics: Mechanisms and lab approaches. Cancer Lett..

[28] Grogan T.M., Spier C.M., Salmon S.E., Matzner M., Rybski J., Weinstein R.S., Scheper R.J., Dalton W.S. (1993). P-glycoprotein expression in human plasma cell myeloma: Correlation with prior chemotherapy. Blood.

[29] Chernykh Y., Golenkov A., Vysotskaya L., Shushanov S., Rybalkina E. (2016). Effect of Expression of Multidrug Resistance Genes in Newly Diagnosed Multiple Myeloma on the Clinical Course of the Disease. Blood.

[30] Zhou W., Yang Y., Xia J., Wang H., Salama M.E., Xiong W., Xu H., Shetty S., Chen T., Zeng Z. (2013). NEK2 induces drug resistance mainly through activation of efflux drug pumps and is associated with poor prognosis in myeloma and other cancers. Cancer Cell.

[31] Ishikawa T., Müller M., Klünemann C., Schaub T., Keppler D. (1990). ATP-dependent primary active transport of cysteinyl leukotrienes across liver canalicular membrane. Role of the ATP-dependent transport system for glutathione S-conjugates. J. Biol. Chem..

[32] Lagas J.S., Sparidans R.W., Wagenaar E., Beijnen J.H., Schinkel A.H. (2010). Hepatic Clearance of Reactive Glucuronide Metabolites of Diclofenac in the Mouse Is Dependent on Multiple ATP-Binding Cassette Efflux Transporters. Mol. Pharmacol..

[33] Sassi Y., Lipskaia L., Vandecasteele G., Nikolaev V.O., Hatem S.N., Cohen Aubart F., Russel F.G., Mougenot N., Vrignaud C., Lechat P. (2008). Multidrug resistance-associated protein 4 regulates cAMP-dependent signaling pathways and controls human and rat SMC proliferation. J. Clin. Investig..

[34] Kool M., van der Linden M., de Haas M., Baas F., Borst P. (1999). Expression of human MRP6, a homologue of the multidrug resistance protein gene MRP1, in tissues and cancer cells. Cancer Res..

[35] Kozalak G., Oksuzoglu E. (2021). Efficacy of Multi-Drug Resistance Transporters and Glutathione S-Transferase P-1 at Developing Bortezomib Resistance in Multiple Myeloma Cell Lines. Lat. Am. J. Pharm..

[36] Guo Y., Köck K., Ritter C.A., Chen Z.-S., Grube M., Jedlitschky G., Illmer T., Ayres M., Beck J.F., Siegmund W. (2009). Expression of ABCC-Type Nucleotide Exporters in Blasts of Adult Acute Myeloid Leukemia: Relation to Long-term Survival. Clin. Cancer Res..

[37] Morrow C.S., Smitherman P.K., Townsend A.J. (1998). Combined expression of multidrug resistance protein (MRP) and glutathione S-transferase P1-1 (GSTP1-1) in MCF7 cells and high level resistance to the cytotoxicities of ethacrynic acid but not oxazaphosphorines or cisplatin. Biochem. Pharmacol..

[38] Zhao J., Wang M., He P., Chen Y., Wang X., Zhang M. (2020). Identification of glutathione S-transferase π 1 as a prognostic proteomic biomarker for multiple myeloma using proteomic profiling. Oncol. Lett..

[39] Soriano G.P., Besse L., Li N., Kraus M., Besse A., Meeuwenoord N., Bader J., Everts B., den Dulk H., Overkleeft H.S. (2016). Proteasome inhibitor-adapted myeloma cells are largely independent from proteasome activity and show complex proteomic changes, in particular in redox and energy metabolism. Leukemia.

[40] Manik C., Mindaugas A., Thorsten S., Elisabeth M., Claudia H., Torsten S., Tanja H., Heike S., Stefanie K., Hermann E. (2013). The PI3K/Akt signaling pathway regulates the expression of Hsp70, which critically contributes to Hsp90-chaperone function and tumor cell survival in multiple myeloma. Haematologica.

[41] Franke N.E., Niewerth D., Assaraf Y.G., van Meerloo J., Vojtekova K., van Zantwijk C.H., Zweegman S., Chan E.T., Kirk C.J., Geerke D.P. (2012). Impaired bortezomib binding to mutant β5 subunit of the proteasome is the underlying basis for bortezomib resistance in leukemia cells. Leukemia.

[42] Suzuki E., Demo S., Deu E., Keats J., Arastu-Kapur S., Bergsagel P.L., Bennett M.K., Kirk C.J. (2011). Molecular mechanisms of bortezomib resistant adenocarcinoma cells. PLoS ONE.

[43] Abdel-Wahab A.F., Mahmoud W., Al-Harizy R.M. (2019). Targeting glucose metabolism to suppress cancer progression: Prospective of anti-glycolytic cancer therapy. Pharmacol. Res..

[44] Sekiguchi N., Ootsubo K., Wagatsuma M., Midorikawa K., Nagata A., Noto S., Yamada K., Takezako N. (2014). The impact of C-Myc gene-related aberrations in newly diagnosed myeloma with bortezomib/dexamethasone therapy. Int. J. Hematol..

[45] Robak P., Drozdz I., Szemraj J., Robak T. (2018). Drug resistance in multiple myeloma. Cancer Treat. Rev..

[46] Öksüzoğlu E., Kozalak G. (2021). Inhibition of apoptosis may lead to the development of bortezomib resistance in multiple myeloma cancer cells. Turk. J. Biochem..

[47] Spets H., Strömberg T., Georgii-Hemming P., Siljason J., Nilsson K., Jernberg-Wiklund H. (2002). Expression of the bcl-2 family of pro- and anti-apoptotic genes in multiple myeloma and normal plasma cells: Regulation during interleukin-6(IL-6)-induced growth and survival. Eur. J. Haematol..

[48] Neri P., Gratton K., Ren L., Mansoor A., Duggan P., Stewart D.A., Bahlis N.J. (2009). miRNA Expression in Multiple Myeloma as Predictive Model of Response to Bortezomib. Blood.

[49] Chauhan D., Hideshima T., Anderson K.C. (2005). Proteasome inhibition in multiple myeloma: Therapeutic implication. Annu. Rev. Pharmacol. Toxicol..

[50] Cusack J.C. (2003). Rationale for the treatment of solid tumors with the proteasome inhibitor bortezomib. Cancer Treat. Rev..

[51] Schmidmaier R., Mörsdorf K., Baumann P., Emmerich B., Meinhardt G. (2006). Evidence for cell adhesion-mediated drug resistance of multiple myeloma cells in vivo. Int. J. Biol. Markers.

[52] Cencini E., Fabbri A., Sicuranza A., Gozzetti A., Bocchia M. (2021). The Role of Tumor-Associated Macrophages in Hematologic Malignancies. Cancers.

[53] Ahmed S., Khan H., Aschner M., Mirzae H., Küpeli Akkol E., Capasso R. (2020). Anticancer Potential of Furanocoumarins: Mechanistic and Therapeutic Aspects. Int. J. Mol. Sci..

[54] Robey R.W., Pluchino K.M., Hall M.D., Fojo A.T., Bates S.E., Gottesman M.M. (2018). Revisiting the role of ABC transporters in multidrug-resistant cancer. Nat. Rev. Cancer.

[55] Waghray D., Zhang Q. (2018). Inhibit or Evade Multidrug Resistance P-Glycoprotein in Cancer Treatment. J. Med. Chem..

[56] Juliano R.L., Ling V. (1976). A surface glycoprotein modulating drug permeability in Chinese hamster ovary cell mutants. Biochim. Biophys Acta.

[57] Christie E.L., Pattnaik S., Beach J., Copeland A., Rashoo N., Fereday S., Hendley J., Alsop K., Brady S.L., Lamb G. (2019). Multiple ABCB1 transcriptional fusions in drug resistant high-grade serous ovarian and breast cancer. Nat. Commun..

[58] Fletcher J.I., Williams R.T., Henderson M.J., Norris M.D., Haber M. (2016). ABC transporters as mediators of drug resistance and contributors to cancer cell biology. Drug Resist. Updates Rev. Comment. Antimicrob. Anticancer Chemother..

[59] Verbrugge S.E., Assaraf Y.G., Dijkmans B.A., Scheffer G.L., Al M., den Uyl D., Oerlemans R., Chan E.T., Kirk C.J., Peters G.J. (2012). Inactivating PSMB5 Mutations and P-Glycoprotein (Multidrug Resistance-Associated Protein/ATP-Binding Cassette B1) Mediate Resistance to Proteasome Inhibitors: Ex Vivo Efficacy of (Immuno)Proteasome Inhibitors in Mononuclear Blood Cells from Patients with Rheumatoid Arthritis. J. Pharmacol. Exp. Ther..

[60] O’Connor R., Ooi M.G., Meiller J., Jakubikova J., Klippel S., Delmore J., Richardson P., Anderson K., Clynes M., Mitsiades C.S. (2013). The interaction of bortezomib with multidrug transporters: Implications for therapeutic applications in advanced multiple myeloma and other neoplasias. Cancer Chemother. Pharmacol..

[61] Doyle L.A., Yang W., Abruzzo L.V., Krogmann T., Gao Y., Rishi A.K., Ross D.D. (1998). A multidrug resistance transporter from human MCF-7 breast cancer cells. Proc. Natl. Acad. Sci. USA.

[62] Maliepaard M., Scheffer G.L., Faneyte I.F., van Gastelen M.A., Pijnenborg A.C., Schinkel A.H., van De Vijver M.J., Scheper R.J., Schellens J.H. (2001). Subcellular localization and distribution of the breast cancer resistance protein transporter in normal human tissues. Cancer Res..

[63] Mo W., Zhang J.T. (2012). Human ABCG2: Structure, function, and its role in multidrug resistance. Int. J. Biochem. Mol. Biol..

[64] Turner J.G., Gump J.L., Zhang C., Cook J.M., Marchion D., Hazlehurst L., Munster P., Schell M.J., Dalton W.S., Sullivan D.M. (2006). ABCG2 expression, function, and promoter methylation in human multiple myeloma. Blood.

[65] Scheffer G.L., Wijngaard P.L.J., Flens M.J., Izquierdo M.A., Slovak M.L., Pinedo H.M., Meijer C.J.L.M., Clevers H.C., Scheper R.J. (1995). The drug resistance-related protein LRP is the human major vault protein. Nat. Med..

[66] Krishnan S.R., Jaiswal R., Brown R.D., Luk F., Bebawy M. (2016). Multiple myeloma and persistence of drug resistance in the age of novel drugs (Review). Int. J. Oncol..

[67] Kulsoom B., Shamsi T.S., Afsar N.A. (2019). Lung resistance-related protein (LRP) predicts favorable therapeutic outcome in Acute Myeloid Leukemia. Sci. Rep..

[68] Huh H.J., Park C.-J., Jang S., Seo E.-J., Chi H.-S., Lee J.-H., Lee K.-H., Seo J.J., Moon H.N., Ghim T. (2006). Prognostic Significance of Multidrug Resistance Gene 1 (MDR1), Multidrug Resistance-related Protein (MRP) and Lung Resistance Protein (LRP) mRNA Expression in Acute Leukemia. J. Korean Med. Sci..

[69] Mándoky L., Géczi L., Doleschall Z., Bodrogi I., Csuka O., Kásler M., Bak M. (2004). Expression and prognostic value of the lung resistance-related protein (LRP) in germ cell testicular tumors. Anticancer Res..

[70] Burger H., Foekens J.A., Look M.P., Meijer-van Gelder M.E., Klijn J.G., Wiemer E.A., Stoter G., Nooter K. (2003). RNA expression of breast cancer resistance protein, lung resistance-related protein, multidrug resistance-associated proteins 1 and 2, and multidrug resistance gene 1 in breast cancer: Correlation with chemotherapeutic response. Clin. Cancer Res..

[71] Raaijmakers H.G., Izquierdo M.A., Lokhorst H.M., de Leeuw C., Belien J.A., Bloem A.C., Dekker A.W., Scheper R.J., Sonneveld P. (1998). Lung-resistance-related protein expression is a negative predictive factor for response to conventional low but not to intensified dose alkylating chemotherapy in multiple myeloma. Blood.

[72] Cole S.P.C., Bhardwaj G., Gerlach J.H., Mackie J.E., Grant C.E., Almquist K.C., Stewart A.J., Kurz E.U., Duncan A.M.V., Deeley R.G. (1992). Overexpression of a transporter gene in a multidrug-resistant human lung cancer cell line. Science.

[73] Johnson Z.L., Chen J. (2017). Structural Basis of Substrate Recognition by the Multidrug Resistance Protein MRP1. Cell.

[74] Kumar A., Jaitak V. (2019). Natural products as multidrug resistance modulators in cancer. Eur. J. Med. Chem..

[75] Loe D.W., Deeley R.G., Cole S.P.C. (1998). Characterization of Vincristine Transport by the Mr 190,000 Multidrug Resistance Protein (MRP): Evidence for Cotransport with Reduced Glutathione1. Cancer Res..

[76] Renes J., de Vries E.G., Nienhuis E.F., Jansen P.L., Müller M. (1999). ATP- and glutathione-dependent transport of chemotherapeutic drugs by the multidrug resistance protein MRP1. Br. J. Pharmacol..

[77] Nasr R., Lorendeau D., Khonkarn R., Dury L., Pérès B., Boumendjel A., Cortay J.C., Falson P., Chaptal V., Baubichon-Cortay H. (2020). Molecular analysis of the massive GSH transport mechanism mediated by the human Multidrug Resistant Protein 1/ABCC1. Sci. Rep..

[78] Hopper-Borge E., Xu X., Shen T., Shi Z., Chen Z.-S., Kruh G.D. (2008). Human Multidrug Resistance Protein 7 (ABCC10) Is a Resistance Factor for Nucleoside Analogues and Epothilone B. Cancer Res..

[79] Sarkadi B., Homolya L., Szakács G., Váradi A. (2006). Human multidrug resistance ABCB and ABCG transporters: Participation in a chemoimmunity defense system. Physiol. Rev..

[80] Petrini M., Di Simone D., Favati A., Mattii L., Valentini P., Grassi B. (1995). GST-pi and P-170 co-expression in multiple myeloma. Br. J. Haematol..

[81] Ishikawa T. (1992). The ATP-dependent glutathione S-conjugate export pump. Trends Biochem. Sci..

[82] Zaman G.J., Lankelma J., Tellingen O.V., Beijnen J., Dekker H., Paulusma C., Elferink R.P.O., Baas F., Borst P. (1995). Role of glutathione in the export of compounds from cells by the multidrug-resistance-associated protein. Proc. Natl. Acad. Sci. USA.

[83] Keppler D., König J. (1997). Expression and localization of the conjugate export pump encoded by the MRP2 (cMRP/cMOAJ) gene in liver. FASEB J..

[84] Armstrong R.N. (1987). Enzyme-catalyzed detoxication reactions: Mechanisms and stereochemistry. CRC Crit. Rev. Biochem..

[85] Sau A., Pellizzari Tregno F., Valentino F., Federici G., Caccuri A.M. (2010). Glutathione transferases and development of new principles to overcome drug resistance. Arch. Biochem. Biophys..

[86] Tew K.D., Dutta S., Schultz M. (1997). Inhibitors of glutathione S-transferases as therapeutic agents. Adv. Drug Deliv. Rev..

[87] Holkova B., Grant S. (2012). Proteasome inhibitors in mantle cell lymphoma. Best Pract. Res. Clin. Haematol..

[88] Obeng E.A., Carlson L.M., Gutman D.M., Harrington W.J., Lee K.P., Boise L.H. (2006). Proteasome inhibitors induce a terminal unfolded protein response in multiple myeloma cells. Blood.

[89] Egan P., Drain S., Conway C., Bjourson A.J., Alexander H.D. (2016). Towards Stratified Medicine in Plasma Cell Myeloma. Int. J. Mol. Sci..

[90] Nikesitch N., Ling S.C. (2016). Molecular mechanisms in multiple myeloma drug resistance. J. Clin. Pathol..

[91] Yun Z., Zhichao J., Hao Y., Ou J., Ran Y., Wen D., Qun S. (2017). Targeting autophagy in multiple myeloma. Leuk. Res..

[92] Amaravadi R.K., Lippincott-Schwartz J., Yin X.M., Weiss W.A., Takebe N., Timmer W., DiPaola R.S., Lotze M.T., White E. (2011). Principles and current strategies for targeting autophagy for cancer treatment. Clin. Cancer Res..

[93] Milani M., Rzymski T., Mellor H.R., Pike L., Bottini A., Generali D., Harris A.L. (2009). The Role of ATF4 Stabilization and Autophagy in Resistance of Breast Cancer Cells Treated with Bortezomib. Cancer Res..

[94] Huber E.M., Heinemeyer W., Groll M. (2015). Bortezomib-resistant mutant proteasomes: Structural and biochemical evaluation with carfilzomib and ONX 0914. Structure.

[95] Kubiczkova L., Pour L., Sedlarikova L., Hajek R., Sevcikova S. (2014). Proteasome inhibitors—Molecular basis and current perspectives in multiple myeloma. J. Cell. Mol. Med..

[96] Johnsen A., France J., Sy M.S., Harding C.V. (1998). Down-regulation of the transporter for antigen presentation, proteasome subunits, and class I major histocompatibility complex in tumor cell lines. Cancer Res..

[97] Kitamura A., Maekawa Y., Uehara H., Izumi K., Kawachi I., Nishizawa M., Toyoshima Y., Takahashi H., Standley D.M., Tanaka K. (2011). A mutation in the immunoproteasome subunit PSMB8 causes autoinflammation and lipodystrophy in humans. J. Clin. Investig..

[98] Furukawa Y., Kikuchi J. (2020). Molecular basis of clonal evolution in multiple myeloma. Int. J. Hematol..

[99] Morgan G.J., Walker B.A., Davies F.E. (2012). The genetic architecture of multiple myeloma. Nat. Rev. Cancer.

[100] Solary E., Droin N., Bettaieb A., Corcos L., Dimanche-Boitrel M.T., Garrido C. (2000). Positive and negative regulation of apoptotic pathways by cytotoxic agents in hematological malignancies. Leukemia.

[101] Abdi J., Chen G., Chang H. (2013). Drug resistance in multiple myeloma: Latest findings and new concepts on molecular mechanisms. Oncotarget.

[102] Mitsiades N., Mitsiades C.S., Poulaki V., Chauhan D., Fanourakis G., Gu X., Bailey C., Joseph M., Libermann T.A., Treon S.P. (2002). Molecular sequelae of proteasome inhibition in human multiple myeloma cells. Proc. Natl. Acad. Sci. USA.

[103] Balsas P., López-Royuela N., Galán-Malo P., Anel A., Marzo I., Naval J. (2009). Cooperation between Apo2L/TRAIL and bortezomib in multiple myeloma apoptosis. Biochem. Pharmacol..

[104] Liu J., Hamrouni A., Wolowiec D., Coiteux V., Kuliczkowski K., Hetuin D., Saudemont A., Quesnel B. (2007). Plasma cells from multiple myeloma patients express B7-H1 (PD-L1) and increase expression after stimulation with IFN-{gamma} and TLR ligands via a MyD88-, TRAF6-, and MEK-dependent pathway. Blood.

[105] Xie T., Geng J., Wang Y., Wang L., Huang M., Chen J., Zhang K., Xue L., Liu X., Mao X. (2017). FOXM1 evokes 5-fluorouracil resistance in colorectal cancer depending on ABCC10. Oncotarget.

[106] Townsend D.M., Tew K.D. (2003). The role of glutathione-S-transferase in anti-cancer drug resistance. Oncogene.

[107] Bi C., Chng W.J. (2014). MicroRNA: Important Player in the Pathobiology of Multiple Myeloma. BioMed Res. Int..

[108] Pichiorri F., Suh S.S., Ladetto M., Kuehl M., Palumbo T., Drandi D., Taccioli C., Zanesi N., Alder H., Hagan J.P. (2008). MicroRNAs regulate critical genes associated with multiple myeloma pathogenesis. Proc. Natl. Acad. Sci. USA.

[109] Chen L., Li C., Zhang R., Gao X., Qu X., Zhao M., Qiao C., Xu J., Li J. (2011). miR-17-92 cluster microRNAs confers tumorigenicity in multiple myeloma. Cancer Lett..

[110] Zhang Y.K., Wang H., Leng Y., Li Z.L., Yang Y.F., Xiao F.J., Li Q.F., Chen X.Q., Wang L.S. (2011). Overexpression of microRNA-29b induces apoptosis of multiple myeloma cells through down regulating Mcl-1. Biochem. Biophys. Res. Commun..

[111] Löffler D., Brocke-Heidrich K., Pfeifer G., Stocsits C., Hackermüller J., Kretzschmar A.K., Burger R., Gramatzki M., Blumert C., Bauer K. (2007). Interleukin-6 dependent survival of multiple myeloma cells involves the Stat3-mediated induction of microRNA-21 through a highly conserved enhancer. Blood.

[112] Gururajan M., Haga C.L., Das S., Leu C.M., Hodson D., Josson S., Turner M., Cooper M.D. (2010). MicroRNA 125b inhibition of B cell differentiation in germinal centers. Int. Immunol..

[113] Morelli E., Biamonte L., Federico C., Amodio N., Di Martino M.T., Gallo Cantafio M.E., Manzoni M., Scionti F., Samur M.K., Gullà A. (2018). Therapeutic vulnerability of multiple myeloma to MIR17PTi, a first-in-class inhibitor of pri-miR-17-92. Blood.

[114] Yang W.C., Lin S.F. (2015). Mechanisms of Drug Resistance in Relapse and Refractory Multiple Myeloma. BioMed Res. Int..

[115] Fabre C., Mimura N., Bobb K., Kong S.Y., Gorgun G., Cirstea D., Hu Y., Minami J., Ohguchi H., Zhang J. (2012). Dual inhibition of canonical and noncanonical NF-κB pathways demonstrates significant antitumor activities in multiple myeloma. Clin. Cancer Res..

[116] Markovina S., Callander N.S., O’Connor S.L., Kim J., Werndli J.E., Raschko M., Leith C.P., Kahl B.S., Kim K., Miyamoto S. (2008). Bortezomib-resistant nuclear factor-kappaB activity in multiple myeloma cells. Mol. Cancer Res..

[117] Meads M.B., Gatenby R.A., Dalton W.S. (2009). Environment-mediated drug resistance: A major contributor to minimal residual disease. Nat. Rev. Cancer.

[118] Markovina S., Callander N.S., O’Connor S.L., Xu G., Shi Y., Leith C.P., Kim K., Trivedi P., Kim J., Hematti P. (2010). Bone marrow stromal cells from multiple myeloma patients uniquely induce bortezomib resistant NF-kappaB activity in myeloma cells. Mol. Cancer.

[119] Yang Y., Chen Y., Saha M.N., Chen J., Evans K., Qiu L., Reece D., Chen G.A., Chang H. (2015). Targeting phospho-MARCKS overcomes drug-resistance and induces antitumor activity in preclinical models of multiple myeloma. Leukemia.

[120] Wang J., Hendrix A., Hernot S., Lemaire M., De Bruyne E., Van Valckenborgh E., Lahoutte T., De Wever O., Vanderkerken K., Menu E. (2014). Bone marrow stromal cell–derived exosomes as communicators in drug resistance in multiple myeloma cells. Blood.

[121] Yang Y., Shi J., Tolomelli G., Xu H., Xia J., Wang H., Zhou W., Zhou Y., Das S., Gu Z. (2013). RARα2 expression confers myeloma stem cell features. Blood.

[122] Federico C., Alhallak K., Sun J., Duncan K., Azab F., Sudlow G.P., de la Puente P., Muz B., Kapoor V., Zhang L. (2020). Tumor microenvironment-targeted nanoparticles loaded with bortezomib and ROCK inhibitor improve efficacy in multiple myeloma. Nat. Commun..

[123] Omstead D.T., Mejia F., Sjoerdsma J., Kim B., Shin J., Khan S., Wu J., Kiziltepe T., Littlepage L.E., Bilgicer B. (2020). In vivo evaluation of CD38 and CD138 as targets for nanoparticle-based drug delivery in multiple myeloma. J. Hematol. Oncol..

[124] de la Puente P., Luderer M.J., Federico C., Jin A., Gilson R.C., Egbulefu C., Alhallak K., Shah S., Muz B., Sun J. (2018). Enhancing proteasome-inhibitory activity and specificity of bortezomib by CD38 targeted nanoparticles in multiple myeloma. J. Control. Release.

[125] Qu Y., Chu B., Wei X., Chen Y., Yang Y., Hu D., Huang J., Wang F., Chen M., Zheng Y. (2022). Cancer-Cell-Biomimetic Nanoparticles for Targeted Therapy of Multiple Myeloma Based on Bone Marrow Homing. Adv. Mater..

[126] Hu X., Chai Z., Lu L., Ruan H., Wang R., Zhan C., Xie C., Pan J., Liu M., Wang H. (2019). Bortezomib Dendrimer Prodrug-Based Nanoparticle System. Adv. Funct. Mater..

[127] Gu Z., Wang X., Cheng R., Cheng L., Zhong Z. (2018). Hyaluronic acid shell and disulfide-crosslinked core micelles for in vivo targeted delivery of bortezomib for the treatment of multiple myeloma. Acta Biomater..

[128] Nigro A., Frattaruolo L., Fava M., De Napoli I., Greco M., Comandè A., De Santo M., Pellegrino M., Ricci E., Giordano F. (2020). Bortezomib-Loaded Mesoporous Silica Nanoparticles Selectively Alter Metabolism and Induce Death in Multiple Myeloma Cells. Cancers.

[129] Patra C.R., Verma R., Kumar S., Greipp P.R., Mukhopadhyay D., Mukherjee P. (2008). Fabrication of Gold Nanoparticle for Potential Application in Multiple Myeloma. J. Biomed. Nanotechnol..

[130] Che F., Chen J., Dai J., Liu X. (2020). Inhibition of Multiple Myeloma Using 5-Aza-2′-Deoxycytidine and Bortezomib-Loaded Self-Assembling Nanoparticles. Cancer Manag. Res..

[131] Waldschmidt J.M., Fruttiger S.J., Wider D., Jung J., Thomsen A.R., Hartmann T.N., Duyster J., Hug M.J., Azab K.A., Jung M. (2022). Ex vivo propagation in a novel 3D high-throughput co-culture system for multiple myeloma. J. Cancer Res. Clin. Oncol..

[132] Wu D., Wang Z., Li J., Song Y., Perez M.E.M., Wang Z., Cao X., Cao C., Maharjan S., Anderson K.C. (2022). A 3D-Bioprinted Multiple Myeloma Model. Adv. Healthc. Mater..

[133] Clara-Trujillo S., Tolosa L., Cordón L., Sempere A., Gallego Ferrer G., Gómez Ribelles J.L. (2022). Novel microgel culture system as semi-solid three-dimensional in vitro model for the study of multiple myeloma proliferation and drug resistance. Biomater. Adv..

[134] Li Y.-R., Yu Y., Kramer A., Hon R., Wilson M., Brown J., Yang L. (2022). An Ex Vivo 3D Tumor Microenvironment-Mimicry Culture to Study TAM Modulation of Cancer Immunotherapy. Cells.

[135] Reagan M.R., Mishima Y., Glavey S.V., Zhang Y., Manier S., Lu Z.N., Memarzadeh M., Zhang Y., Sacco A., Aljawai Y. (2014). Investigating osteogenic differentiation in multiple myeloma using a novel 3D bone marrow niche model. Blood.

[136] Freitas Misakyan M.F., Wijeratne E.M.K., Issa M.E., Xu Y.-M., Monteillier A., Gunatilaka A.A.L., Cuendet M. (2021). Structure–Activity Relationships of Withanolides as Antiproliferative Agents for Multiple Myeloma: Comparison of Activity in 2D Models and a 3D Coculture Model. J. Nat. Prod..

[137] Jakubikova J., Cholujova D., Hideshima T., Gronesova P., Soltysova A., Harada T., Joo J., Kong S.Y., Szalat R.E., Richardson P.G. (2016). A novel 3D mesenchymal stem cell model of the multiple myeloma bone marrow niche: Biologic and clinical applications. Oncotarget.

[138] Sui C., Zilberberg J., Lee W. (2022). Microfluidic device engineered to study the trafficking of multiple myeloma cancer cells through the sinusoidal niche of bone marrow. Sci. Rep..

[139] Ouyang D., Li Y., He W., Lin W., Hu L., Wang C., Xu L., Park J., You L. (2019). Mechanical segregation and capturing of clonal circulating plasma cells in multiple myeloma using micropillar-integrated microfluidic device. Biomicrofluidics.

[140] Carreras P., Gonzalez I., Gallardo M., Ortiz-Ruiz A., Martinez-Lopez J. (2020). Droplet Microfluidics for the ex Vivo Expansion of Human Primary Multiple Myeloma Cells. Micromachines.

[141] Moore T.A., Brodersen P., Young E.W.K. (2017). Multiple Myeloma Cell Drug Responses Differ in Thermoplastic vs. PDMS Microfluidic Devices. Anal. Chem..

[142] Zeng Y., Gao L., Luo X., Chen Y., Kabeer M.H., Chen X., Stucky A., Loudon W.G., Li S.C., Zhang X. (2018). Microfluidic enrichment of plasma cells improves treatment of multiple myeloma. Mol. Oncol..

[143] Pak C., Callander N.S., Young E.W., Titz B., Kim K., Saha S., Chng K., Asimakopoulos F., Beebe D.J., Miyamoto S. (2015). MicroC(3): An ex vivo microfluidic cis-coculture assay to test chemosensitivity and resistance of patient multiple myeloma cells. Integr. Biol..

[144] Glaser D.E., Curtis M.B., Sariano P.A., Rollins Z.A., Shergill B.S., Anand A., Deely A.M., Shirure V.S., Anderson L., Lowen J.M. (2022). Organ-on-a-chip model of vascularized human bone marrow niches. Biomaterials.

[145] Yetisgin A.A., Cetinel S., Zuvin M., Kosar A., Kutlu O. (2020). Therapeutic Nanoparticles and Their Targeted Delivery Applications. Molecules.

[146] Iannazzo D., Ettari R., Giofrè S., Eid A.H., Bitto A. (2020). Recent Advances in Nanotherapeutics for Multiple Myeloma. Cancers.

[147] Zheleznyak A., Shokeen M., Achilefu S. (2018). Nanotherapeutics for multiple myeloma. Wiley Interdiscip. Rev. Nanomed. Nanobiotechnology.

[148] Savjani K.T., Gajjar A.K., Savjani J.K. (2012). Drug solubility: Importance and enhancement techniques. Int. Sch. Res. Not. Pharm..

[149] Barenholz Y.C. (2021). Doxil^®^—The first FDA-approved nano-drug: From an idea to a product. Handbook of Harnessing Biomaterials in Nanomedicine.

[150] Patra J.K., Das G., Fraceto L.F., Campos E.V.R., Rodriguez-Torres M.D.P., Acosta-Torres L.S., Diaz-Torres L.A., Grillo R., Swamy M.K., Sharma S. (2018). Nano based drug delivery systems: Recent developments and future prospects. J. Nanobiotechnology.

[151] Lee K.H., Kim T.H. (2021). Recent Advances in Multicellular Tumor Spheroid Generation for Drug Screening. Biosensors.

[152] Regmi S., Poudel C., Adhikari R., Luo K.Q. (2022). Applications of Microfluidics and Organ-on-a-Chip in Cancer Research. Biosensors.

[153] Mehta P., Rahman Z., Ten Dijke P., Boukany P.E. (2022). Microfluidics meets 3D cancer cell migration. Trends Cancer.

[154] Gharib G., Bütün İ., Muganlı Z., Kozalak G., Namlı İ., Sarraf S.S., Ahmadi V.E., Toyran E., van Wijnen A.J., Koşar A. (2022). Biomedical Applications of Microfluidic Devices: A Review. Biosensors.

[155] Young E.W., Pak C., Kahl B.S., Yang D.T., Callander N.S., Miyamoto S., Beebe D.J. (2012). Microscale functional cytomics for studying hematologic cancers. Blood.

[156] Mahmoudian M., Valizadeh H., Löbenberg R., Zakeri-Milani P. (2021). Bortezomib-loaded lipidic-nano drug delivery systems; formulation, therapeutic efficacy, and pharmacokinetics. J. Microencapsul..

[157] Cetin A.E., Stevens M.M., Calistri N.L., Fulciniti M., Olcum S., Kimmerling R.J., Munshi N.C., Manalis S.R. (2017). Determining therapeutic susceptibility in multiple myeloma by single-cell mass accumulation. Nat. Commun..

[158] Sung H.W., Choi S.-E., Chu C.H., Ouyang M., Kalyan S., Scott N., Hur S.C. (2022). Sensitizing drug-resistant cancer cells from blood using microfluidic electroporator. PLoS ONE.

[159] Postek W., Garstecki P. (2022). Droplet Microfluidics for High-Throughput Analysis of Antibiotic Susceptibility in Bacterial Cells and Populations. Acc. Chem. Res..

[160] Bhatia S.N., Ingber D.E. (2014). Microfluidic organs-on-chips. Nat. Biotechnol..

[161] Pires de Mello C.P., Carmona-Moran C., McAleer C.W., Perez J., Coln E.A., Long C.J., Oleaga C., Riu A., Note R., Teissier S. (2020). Microphysiological heart-liver body-on-a-chip system with a skin mimic for evaluating topical drug delivery. Lab Chip.

[162] Maschmeyer I., Lorenz A.K., Schimek K., Hasenberg T., Ramme A.P., Hübner J., Lindner M., Drewell C., Bauer S., Thomas A. (2015). A four-organ-chip for interconnected long-term co-culture of human intestine, liver, skin and kidney equivalents. Lab Chip.

[163] Sung J.H., Wang Y.I., Narasimhan Sriram N., Jackson M., Long C., Hickman J.J., Shuler M.L. (2019). Recent Advances in Body-on-a-Chip Systems. Anal. Chem..

[164] Ma C., Peng Y., Li H., Chen W. (2021). Organ-on-a-Chip: A New Paradigm for Drug Development. Trends Pharmacol. Sci..

[165] Imura Y., Sato K., Yoshimura E. (2010). Micro Total Bioassay System for Ingested Substances: Assessment of Intestinal Absorption, Hepatic Metabolism, and Bioactivity. Anal. Chem..

[166] Nelson M.R., Ghoshal D., Mejías J.C., Rubio D.F., Keith E., Roy K. (2021). A multi-niche microvascularized human bone marrow (hBM) on-a-chip elucidates key roles of the endosteal niche in hBM physiology. Biomaterials.

[167] Galván-Chacón V.P., Zampouka A., Hesse B., Bohner M., Habibovic P., Barata D. (2022). Bone-on-a-Chip: A Microscale 3D Biomimetic Model to Study Bone Regeneration. Adv. Eng. Mater..

[168] Das B., Seesala S.V., Pal P., Roy T., Roy P.G., Dhara S. (2022). A vascularized bone-on-a-chip model development via exploring mechanical stimulation for evaluation of fracture healing therapeutics. Vitr. Model..

[169] Chou D.B., Frismantas V., Milton Y., David R., Pop-Damkov P., Ferguson D., MacDonald A., Vargel Bölükbaşı Ö., Joyce C.E., Moreira Teixeira L.S. (2020). On-chip recapitulation of clinical bone marrow toxicities and patient-specific pathophysiology. Nat. Biomed. Eng..

[170] Chen W., Yang Y., Chen Y., Du F., Zhan H. (2016). Cost-effectiveness of bortezomib for multiple myeloma: A systematic review. Clin. Outcomes Res..

[171] Wang H., Xiao L., Tao J., Srinivasan V., Boyce B.F., Ebetino F.H., Oyajobi B.O., Boeckman R.K., Xing L. (2018). Synthesis of a Bone-Targeted Bortezomib with In Vivo Anti-Myeloma Effects in Mice. Pharmaceutics.

[172] Lourenço D., Lopes R., Pestana C., Queirós A.C., João C., Carneiro E.A. (2022). Patient-Derived Multiple Myeloma 3D Models for Personalized Medicine&mdash; Are We There Yet?. Int. J. Mol. Sci..

